# One Health Investigation of a Household *Salmonella* Thompson Outbreak in Italy: Genomic and Epidemiological Characterization of an Emerging Serotype

**DOI:** 10.3390/pathogens14121285

**Published:** 2025-12-13

**Authors:** Marta Bivona, Andrea Francesco De Bene, Valeria Russini, Maria Laura De Marchis, Ilaria Di Domenico, Francesca Riccardi, Matteo Senese, Laura Gasperetti, Francesca Campeis, Luca Di Blasi, Virginia Carfora, Barbara Middei, Gessica Cordaro, Giuseppe Adreani, Paola Marconi, Teresa Bossù

**Affiliations:** 1 Food Microbiology Unit, Istituto Zooprofilattico Sperimentale del Lazio e della Toscana “M. Aleandri”, 00178 Rome, Italy; marta.bivona-esterno@izslt.it (M.B.); valeria.russini@izslt.it (V.R.); marialaura.demarchis@izslt.it (M.L.D.M.); ilaria.didomenico@izslt.it (I.D.D.); francesca.riccardi@izslt.it (F.R.); teresa.bossu@izslt.it (T.B.); 2 UOT Toscana Nord, Istituto Zooprofilattico Sperimentale del Lazio e della Toscana “M. Aleandri”, 56123 Pisa, Italy; matteo.senese@izslt.it (M.S.); laura.gasperetti@izslt.it (L.G.); francesca.campeis@izslt.it (F.C.); luca.diblasi-esterno@izslt.it (L.D.B.); paola.marconi@izslt.it (P.M.); 3 National Reference Laboratory for Antimicrobial Resistance, General Diagnostics Department, Istituto Zooprofilattico Sperimentale del Lazio e della Toscana “M. Aleandri”, 00178 Rome, Italy; virginia.carfora@izslt.it (V.C.); barbara.middei-esterno@izslt.it (B.M.); gessica.cordaro@izslt.it (G.C.); 4 Usl Toscana Nord Ovest, 56123 Pisa, Italy; giuseppe.adreani@uslnordovest.toscana.it

**Keywords:** *Salmonella* Thompson, One Health, salmonellosis, household transmission, central Italy outbreak, companion animals, animal reservoir, AST, WGS

## Abstract

*Salmonella* is a Gram-negative enteric bacterium responsible for the foodborne and waterborne disease salmonellosis, which was the second most reported foodborne gastrointestinal infection in humans in the European Union in 2023. Animals represent the principal reservoir of this pathogen, with animal-derived food products serving as the main route of transmission to humans. In a household context, having numerous animals can be a crucial factor for contracting *Salmonella* spp. infection. In the present study, we report a case of a familiar outbreak of *Salmonella* Thompson that occurred in 2024 in central Italy, involving an infant and the companion animals (a dog, a cat and ten birds) of the family’s farm. To support the epidemiological investigations, antimicrobial susceptibility testing and whole-genome sequencing (WGS) were conducted on strains from the human case and from animals. Eleven strains were isolated in total, from fecal samples collected from the child and the animals at different times. WGS confirmed the genetic relatedness between human and animal isolates, supporting the hypothesis of a shared source of infection, but genes or plasmid involved in antibiotic resistance were not found. Moreover, AST revealed that isolates were fully susceptible to major antimicrobial classes tested. Despite being an uncommon serotype, the involved *Salmonella* Thompson serovar 6,7: k:1,5 O:7 (C1) demonstrated a high pathogenic potential, emphasizing the need for vigilance even toward serotypes not typically associated with major public health concerns. Moreover, these findings underscore the critical need for an integrated One Health approach to effectively monitor, prevent, and control zoonotic infections.

## 1. Introduction

*Salmonella enterica* is a Gram-negative, facultative anaerobic, rod-shaped bacterium belonging to the *Enterobacteriaceae* family responsible for the disease called salmonellosis, which is the second most reported foodborne gastrointestinal infection in humans in the European Union, with 77,486 confirmed cases (18.0 cases per 100,000 population) in 2023 [1,2].

The serotypes most frequently reported as responsible for cases of human salmonellosis were *Salmonella enterica* subsp. *enterica* serovar Enteritidis (70.8%), *S.* Typhimurium (8.9%), *S.* Typhimurium monophasic (5.1%) and *S.* Infantis (2.0%) [2].

Human infection with *Salmonella* can occur through multiple transmission routes, most commonly via the consumption of contaminated food, such as raw or undercooked eggs, insufficiently cooked meat (especially poultry), leafy vegetables (e.g., arugula), or contaminated drinking water [3,4,5]. However, direct or indirect contact with animals carrying the pathogen also represents a significant risk factor, particularly in domestic settings where hygiene measures may be inadequate [6].

*Salmonella* is an enteric pathogen that colonizes the intestinal tract of a wide range of animals, including livestock, birds, pets and wild fauna. Infected animals often remain asymptomatic but can shed high loads of *Salmonella* in their feces, thereby contaminating the environment, water sources, and foodstuffs, in which it can survive for a long period [7].

*S.* Enteriditis and *S*. Typhimurium are the serotypes most associated with human infections and thus prioritized in epidemiological surveillance. However less frequent serotypes such as *S.* Thompson can also be detected in some clinical and environmental contexts [5,8].

*S.* Thompson has been primarily associated with foodborne outbreaks (0.57% of confirmed human salmonellosis cases across 13 member States of the EU in 2023) [2]. However, significant epidemic outbreaks or isolated household cases attributable to infections with this serotype are rarely reported in the literature and therefore deserve attention.

Human health is deeply interconnected with the health of animals and with the environment, forming a delicate equilibrium that emerging epidemics have increasingly highlighted. The rising emergence and spread of zoonotic diseases represent one of the major global health threats today [9]. Therefore, adopting an integrated health framework is essential. The One Health approach meets this need by fostering collaboration across sectors responsible for human, animal (either domestic and wildlife), and environmental health. Implemented at various governance levels, this interdisciplinary and multisectoral strategy enhances health outcomes by acknowledging the interconnected nature of people, animals, plants, and their shared environments [10].

In this study, we present the case of a household infection with *S*. Thompson in central Italy, involving a two-month-old infant and several companion animals. The household animals included one dog, one cat, and ten birds, specifically three peacocks, two guineafowls, one pheasant, and four hens. Over the course of several months of investigation on this family case, microbiological analyses were performed on fecal samples collected from both the child and the animals involved, with the aim of identifying and serotyping the isolated bacterial species, as well as determining their antimicrobial resistance profiles. Furthermore, Whole-Genome Sequencing (WGS) was conducted to investigate the genetic relatedness and epidemiological clustering of selected isolates and for in-depth molecular characterization.

## 2. Materials and Methods

### 2.1. Microbiological Methods for Bacterial Identification and Serotyping

#### 2.1.1. Animals Sample Collection

The veterinary service of the local health authority, together with the freelance veterinarian of the family, was responsible for the collection of animals’ fecal samples as part of the investigation on the household outbreak. Sampling other matrices besides feces, such as food and/or environmental swabs, was not planned for the purposes of this investigation. A total of four sampling rounds were conducted between October 2024 and April 2025, approximately one month apart according to the sampling scheme reported in Table 1. The cat’s feces were sampled only at the last round. During the epidemiological investigation, animals were hosted in the house (one dog and one cat) and in an aviary in the garden of the house (ten birds). Bird feces were taken from the ground of the aviary as a mixture (pooled samples).

#### 2.1.2. Human Sample Collection

The parents of the 2-month-old infant were responsible for collecting the fecal samples. A total of five human feces samples were obtained, with collection dates ranging from October 2024 to June 2025 (Table 1). Microbiological analyses of the child’s fecal samples and *Salmonella* spp. identification and isolation were performed by private or public laboratories.

#### 2.1.3. Microbiological Methods for *Salmonella* Identification

The identification and isolation of *Salmonella* spp. from animal feces were performed according to the OIE method [11] at Istituto Zooprofilattico Sperimentale del Lazio e della Toscana (IZSLT), Territorial Operative Unit (UOT) Tuscany North (Pisa, Italy). The OIE method was used because it is more suitable for samples from animal organs, tissues, and excretions, unlike the ISO 6579-1:2017 + Amd 1:2020 method [12], which is applicable to samples from the primary production stage. Briefly, after pre-enrichment of the samples in Buffered Peptone Water (BPW), selective enrichment was performed in modified semi-solid Rappaport-Vassiliadis (MSRV) Agar, and inoculation of the colonies in Xylose-Lysine-Desoxycholate (XLD) Agar and Shigella Salmonella Agar plates. Biochemical confirmation of *Salmonella* spp. was performed according to the OIE method, following procedures analogous to those described in ISO 6579-1:2017 + Amd 1:2020. Therefore, confirmation was carried out both by macro-method, using TSI agar, Urea agar, and L-Lysine Decarboxylase tests, and by micro-method, using the API 20E system (bioMérieux, Marcy-l’Étoile, France).

All the isolated strains, both from animals and from the infant, were sent to Regional Reference Center for Listeria monocytogenes (CRRLm) of the Pisa Territorial Operations Unit and, subsequently, to the Enteropathogenic bacteria Regional Reference Center (CREP) laboratory of Istituto Zooprofilattico Sperimentale del Lazio e della Toscana (IZSLT), Food Microbiology Unit (Rome, Italy), according to the prescribed sample analysis workflow described by Russini et al. [13], to perform molecular typing, serological typing and sequencing, respectively.

#### 2.1.4. Serotyping

Molecular typing was performed by CRRLm laboratory using Check & Trace Salmonella 2.0 (CTS 2.0) (Check-Points B.V., Wageningen, The Netherlands; Microval certificate 2021LR107_CTS 2.0), a validated molecular-based method for automated identification of *Salmonella* serovars. Serological typing was performed by CREP laboratory according to ISO/TR 6579–3:2014 [14], which involves serological agglutination using specific antisera to identify *Salmonella* O (somatic) and H (flagellar) antigens (Sifin Diagnostics GmbH, Berlin, Germany; SSI Diagnostica A/S, Hillerød, Denmark; Bio-Rad, Hercules, CA, USA).

#### 2.1.5. Antimicrobial Susceptibility Testing (AST)

Antimicrobial susceptibility testing (AST) was performed at the National Reference Laboratory for Antimicrobial Resistance (NRL-AR), Department of General Diagnostics, IZSLT, through minimum inhibitory concentration (MIC) determination by broth microdilution according to the reference method ISO 20776-1:2019 [15], as indicated in the technical requirements set out in point 4, Part A of the Annex of Decision EU 2020/1729 (https://eur-lex.europa.eu/legal-content/EN/TXT/PDF/?uri=CELEX:32020D1729 (accessed on 14 August 2025)). The EU consensus 96-well Sensititre “EU Surveillance *Salmonella*/*E. coli* EUVSEC3 AST Plate”(Thermo Fisher Scientific), with antibiotics and dilution ranges complying with the EU harmonized Monitoring program on AMR (first panel, Table 2 of the EU Decision 2020/1729/EU), was used [16]. The following drugs were tested: amikacin, ampicillin, cefotaxime, ceftazidime, meropenem, azithromycin, chloramphenicol, nalidixic acid, ciprofloxacin, colistin, gentamicin, sulfamethoxazole, tetracycline, tigecycline and trimethoprim. *Escherichia coli* ATCC 25922 was used as a quality control strain. Interpretation of MIC values of amikacin, ampicillin, cefotaxime, ceftazidime, meropenem, chloramphenicol, nalidixic acid, ciprofloxacin, gentamicin, tetracycline and trimethoprim was performed according to EUCAST epidemiological cut-off values (ECOFFs) reported in Table 2 of the EU Decision 2020/1729/EU. Azithromycin, colistin, sulfamethoxazole and tigecycline MIC values were interpreted according to the interpretative thresholds (EUCAST or EFSA ECOFFs) reported in Table B.1 of the EFSA manual published in 2025 [16].

### 2.2. Data Collection of Epidemiological Investigation

A questionnaire was prepared to conduct the epidemiological survey, and written informed consent was signed by the parents for the human case involved in this work. Initially, a meeting was organized with the competent health authority for an initial case understanding. Subsequently, another meeting was organized with the child’s parents, their freelance veterinarian and veterinarians from the competent health authority to gather more comprehensive information and receive direct feedback from the family. The questions referred to the habits of the family and the pets in the household, the feeding habits of all the animals and the details of the symptoms and medical treatment undergone for both child and pets.

### 2.3. Whole-Genome Sequencing and In Silico Analysis

DNA extraction, library preparation, and Illumina sequencing (WGS) were performed for selected isolates as described by De Bene et al. [17] The obtained raw reads were stored in the Sequence Read Archive (SRA) at the GenBank database (NCBI) under the BioProject PRJNA1311533, BioSamples from SAMN50810632 to SAMN50810635. The quality assessment, trimming, and de novo assembly were performed as described by De Bene and colleagues [17]. The genetic relatedness of the isolates was assessed by core genome Multilocus Sequence Typing (cgMLST) analysis, using chewBBACA (v2.8.5) [18] based on the Enterobase cgMLST v2 scheme [19,20]. The MSTreeV2 algorithm in the GrapeTree (v 1.5.0) software was used to visualize the Minimum spanning tree of the cgMLST profiles [21]. A representative isolate was subsequently compared with the *Salmonella* Thompson strains exhibiting the most similar cgMLST profiles available in the international public *Salmonella* database hosted by EnteroBase [22] which includes over 730,000 assembled genomes. The distances were calculated with GrapeTree [21] using the algorithm MSTree V2 for cgMLST allelic distances.

Molecular characterization was performed at the NRL-AR, Department of General Diagnostics, on assembled genomes with the MLST tool for Multilocus Sequence Typing (https://github.com/tseemann/mlst, accessed on 14 August 2025), sistr_cmd v.1.1.3 for in silico serotyping (https://github.com/phac-nml/sistr_cmd, accessed on 14 August 2025) and with the online version of SPIFinder 2.0 (https://cge.food.dtu.dk/services/SPIFinder/, accessed on 14 August 2025) for the identification of *Salmonella* Pathogenicity Islands (SPIs). The ABRicate tool (https://github.com/tseemann/abricate, accessed on 14 August 2025) was also used with the Center for Genomic Epidemiology (CGE) databases (accessed on 14 August 2025) of ResFinder (https://genepi.food.dtu.dk/resfinder, accessed on 14 August 2025) (thresholds of 60% coverage and 90% identity), PlasmidFinder (https://cge.food.dtu.dk/services/PlasmidFinder/, accessed on 14 August 2025) and the virulence factors (VF) database (https://www.mgc.ac.cn/VFs/, accessed on 14 August 2025), for detection of accessory antimicrobial resistance (AMR) genes, plasmid replicons and virulence genes, respectively. The PointFinder-4.1.1 and DisinFinder-2.0.1 databases were also queried to identify, respectively, chromosomal point mutations mediating AMR and acquired disinfectant resistance genes, using the online version of ResFinder-4.7.2 with default parameters (https://genepi.food.dtu.dk/resfinder, accessed on 14 August 2025).

## 3. Results

### 3.1. Case Presentation

In late September 2024 in Tuscany (Central Italy), a 2-month-old infant began to show gastroenteric symptoms, including greenish stools followed by a high fever. The infant was hospitalized, and microbiological testing of the stool confirmed *Salmonella* spp. infection. To assess the extent of potential intra-family transmission, stool samples were also collected from the asymptomatic parents. Both tested negative for *Salmonella* spp.

**Figure 1:**
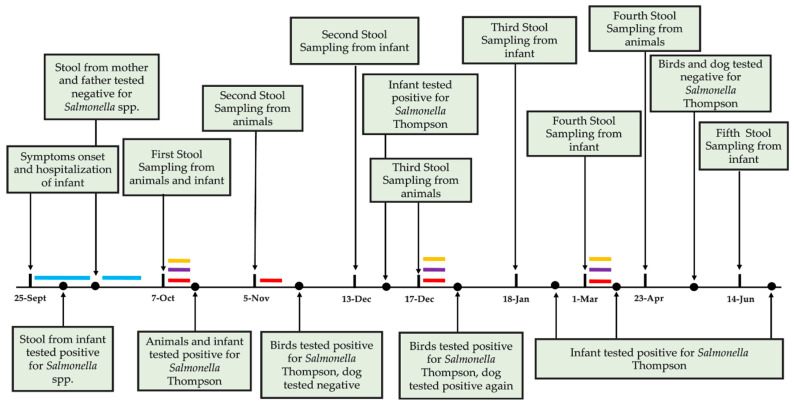
**Timeline of fecal sampling and microbiological test results from the child and household animals from September 2024 to June 2025**. Colored lines indicate orally administered antibiotic treatments. Blue lines = antibiotic treatments administered to the infant: ceftriaxone during hospitalization (25 September 2024–2 October 2024), followed by a short 3-day course of amoxicillin at home. The antibiotic therapy was not prolonged due to the absence of clinical symptoms. Purple and yellow lines = antibiotic treatments administered to the dog and cat, respectively: enrofloxacin for 5 days after the first and third sampling. Red lines = antibiotic treatments administered to the birds: an initial 5-day course of amoxicillin, followed by a 10-day course of trimethosulfa (trimethoprim + sulfadiazine), which continued after the third sampling. The final antibiotic administration for all animals occurred between February and March 2025.

Approximately two weeks after the hospitalization of the infant, the local health authority’s veterinary service started the first fecal sampling of the family’s animals, including one dog (*Canis lupus familiaris*), one cat (*Felis silvestris catus*), three peacocks (*Pavo cristatus*), two guineafowls (*Numida meleagris*), one pheasant (*Phasianus colchius*), and four hens (*Gallus gallus*), while the subsequent samplings were performed by the freelance veterinarian of the family, engaged for the case (Figure 1). The cat’s stools were sampled only during the last collection; both cat and dog, although asymptomatic, were treated with the same antibiotic regimen. All animals showed no symptoms throughout the observation period, except for one leghorn hen, which briefly exhibited signs of illness, including disheveled plumage and a drooping comb. These signs resolved following an additional course of antibiotics.

Investigations conducted by veterinarians of the local health authority and the private veterinarian revealed that the birds had been free roaming in the courtyard prior to the hospitalization of the infant.

Following the confirmed *Salmonella* infection, the birds were confined to their aviary. The only animals always allowed indoors were the dog and the cat, and even then, their access was restricted to areas not frequented by the infant. Household hygiene standards remained consistently adequate, as the competent local health authority reported. The family ceased consuming eggs from their chickens after the outbreak.

Regarding antibiotic treatment, the infant was hospitalized from 25 September to 2 October 2024 and received intravenous ceftriaxone (100 mg) every 12 h. He was also treated with paracetamol and *Lactobacillus reuteri* oral drops for fever and infant gastroenteritis, respectively. After discharge, oral antibiotic therapy continued at home with amoxicillin (2.5 mL every 12 h for three days). Dog and cat received a 5-day course of enrofloxacin following the first positive sampling of the dog, which was then discontinued until after the third sampling, when it was re-administered. The birds were initially treated with a 5-day course of amoxicillin, followed by a 10-day course of trimethoprim-sulfadiazine (trimethosulfa) after the second sampling. Antibiotic therapies on animals were carried out according to the doses recommended by the referring freelance veterinarian for about 20 consecutive days, by oral administration (through drinking water for the birds).

No evidence was found of the consumption of suspicious food or feed, and during the investigation, neither the veterinarians of the local health authority nor the freelance veterinarian of the family collected any samples of household food or feed. The dog and the cat were primarily fed with commercial dry food and were not given raw meat. Birds were fed with a varied diet including cooked meat scraps, raw vegetable leftovers, and commercial feed (occasionally enriched with higher protein content) and rarely bread.

The birds came from different sources. Two peacocks were purchased from a registered breeder in the North of Italy, while a female peafowl hatched at the owner’s residence from eggs discovered incidentally. The Guinea fowl and three hens were obtained from a consortium, whereas a fourth hen hatched at home from fertilized eggs provided by a breeder. The pheasant was acquired from a private breeder.

Other information gathered during the interview revealed that rodent burrows were found in the house’s courtyard, but rodent control measures were carried out. Furthermore, the property birds, since they were housed in their aviary, could not have contact with large avifauna, except for small passerine birds that may enter the aviary. Finally, no wild animals were observed in the property’s courtyard, nor were any large poultry farms located in the vicinity of the house.

### 3.2. Microbiological Analysis and Strains Characterization

The birds tested positive for *Salmonella* spp. during every sampling round until the third one, while the dog alternated between positive and negative results for the same pathogen. All other specimens’ droppings were negative. All animals finally tested negative in the fourth sampling. The infant remained positive at each sampling, even after clinical symptoms had resolved and antibiotic treatment had concluded (Figure 1). Each isolate was identified as *S. enterica* subsp. *enterica* serovar Thompson.

### 3.3. AST

Results of AST were obtained for 10 *S*. Thompson isolates from human and animal sources, including the dog and the birds (Supplementary Table S1). All of them were susceptible to all the tested molecules, except for isolate ID Strain SALM_UM_010325 from the child, which was resistant to ampicillin (MIC value of 32 mg/L).

### 3.4. Whole-Genome Sequencing and In Silico Analysis

**Figure 2:**
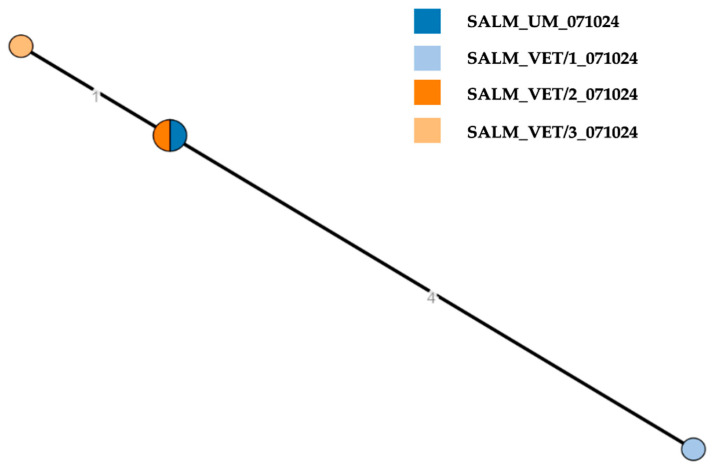
Cluster analysis to assess the genetic relatedness of *Salmonella* Thompson strains isolated from the feces of the child (SALM_UM_071024), the dog (SALM_VET/1_071024), and the pooled fecal samples of the birds (pool 1: SALM_VET/2_071024; pool 2: SALM_VET/3_071024). Pairwise allelic differences ranged from 0 to 5. Allelic distance values are displayed on the branches of the Minimum Spanning Tree.

WGS was performed on a selection of four isolates from representative samples, comprising one isolate from the child, two isolates from the fecal pools taken from the aviary ground and one isolate from the dog (Table 1). The strains resulted in having in silico predicted profile of *S*. Thompson 6,7: k:1,5 O:7 (C1), and all belonging to the Sequence Type (ST)26, Clonal complex (CC)28. The analysis of cgMLST (Figure 2) showed that the four isolates of *S*. Thompson formed a unique cluster, with pairwise allelic distances ranging from 0 to 5.

In the public database Enterobase, we found one Italian isolate belonging to the serotype *S*. Thompson that differs from our cluster by 13 allelic distances. The strain was isolated from the cecal content of broiler chicken sampled at slaughter (North-East Italy) in the frame of the EU Harmonized Monitoring Program on AMR (EU Decision 652/2013/UE) activities, conducted during 2014 in Italy by the NRL-AR, Department of General Diagnostics, IZSLT. In addition, the closest genomes found by core genome MLST analysis were isolates from human cases in the United Kingdom (2014, 2015 and 2024), which differed by 10–17 alleles and human case from France with 16 allelic distances (unknown collection year). Owing to this genetic distance and the lack of any epidemiological link, all these isolates were deemed not relevant to the present study.

### 3.5. Genetic Basis of AMR/Disinfectant Resistance and Plasmid Replicon Typing

The AMR genotypes for the four fully susceptible isolates have been reported in Supplementary Table S1. In detail, all isolates harboured the *aac (6′)-Iaa* gene (WGS-predicted phenotype of ResFinder: amikacin, tobramycin), homologous to *aac (6′)-Iy*, which is cryptic and endogenous in *Salmonella* [23,24] and does not confer resistance to amikacin. All isolates also harboured the *par*C mutation (T57S) which still has a controversial role in conferring (fluoro)quinolone resistance [25]. None of the isolates harboured acquired disinfectant resistance genes or known plasmid replicons.

### 3.6. SPIs and Virulence Genes

Overall SPIs 1, 2, 3, 4, 5, 13, 14, C63PI (SPI-1), CS54 (SPI-24) were identified in the four isolates with a 100% coverage and a range of 98–100% identity. A total of 96 virulence genes belonging to different VF categories (Supplemental Figure S1) were detected in all isolates with 99.5–100% coverage and 96.43–100% identity ranges. Only the *sseI*/*srfH* and *steA* genes were detected with 63.88 and 71.25% coverage values, respectively. In detail, n = 74 genes belonged to the effector delivery-type III secretion system T3SS; n = 21 encoded for fimbrial and non-fimbrial adhesins; n = 2 encoded for nutritional and metabolic factors (MgtBC magnesium transporter system) and also the *mig-14* gene encoding for inner membrane-associated protein, preventing the penetration of antimicrobial peptides (“Antimicrobial activity/Competitive advantage” VF category).

All the SPI sequences detected, except SPI-4, were specifically located in the same contigs of several virulence genes (Table 2).

## 4. Discussion

Companion animals make a significant contribution to human well-being by offering psychological support, companionship, and promoting health-enhancing behaviors. Among these, dogs have been shown to exert a particularly beneficial influence on child development [26].

Pet birds are a smaller and less-known fraction of veterinary patients. Although ornamental birds breeding is a thousand-year-old tradition, the market size has increased significantly in recent years, and globally ornamental birds are gaining popularity as exotic pets because of their high aesthetic value [27,28].

Backyard poultry systems are characterized by private ownership, with products such as eggs and meat typically excluded from commercial markets. Therefore, comprehensive data on owner demographics, husbandry practices, farm structures, and animal welfare are largely unavailable [29]. A review on the backyard poultry system shows that globally many households engage in this type of farming not only for the self-supply of eggs but also for emotional attachment and hobby purposes [30]. This sector represents the fourth tier of poultry production and is distinguished by the lowest standards of biosecurity because of the limited awareness of disease prevention and control among non-professional owners.

A diverse array of animals commonly kept as pets (including reptiles, amphibians, dogs, cats, ornamental birds, and rodents) have the potential to intermittently shed bacteria such as *Salmonella* spp. through their feces, thereby contaminating the household environment. As a result, they are believed to facilitate zoonotic transmission, leading to human salmonellosis [6,31]. Dróżdż et al. [31], in their scientific review, discussed the zoonotic potential of various animal species and the prevalence of *Salmonella* serotypes isolated from different domestic animals worldwide from 2015 to 2021. Regarding birds, including domestic species, several studies have highlighted that direct contact of other animals (such as dogs and cats) with outdoor aviaries may serve as an indirect source of *Salmonella* spp. infection for humans, also considering the bacterium’s ability to persist for extended periods on surfaces such as wood and feces [31]. The same review reported the prevalence of *Salmonella* spp. in domestic dogs and cats. Among dogs, rates of 1.85% were recorded in Spain (2020), 4.9% in the USA (2015), and 12.86% in Thailand (2020). In contrast, the number of reported cases in cats was lower, with prevalences not exceeding 1.77% [31]. Other studies confirmed that, in domestic and public contexts (e.g., veterinarian hospitals), the isolation of *Salmonella* spp. from dogs and cats is frequent, with percentages of 4.4 and 3.7%, respectively [32]. Furthermore, a recent study described an outbreak of *Salmonella enterica* subsp. *enterica* serovar Agona within a Rottweiler breeding kennel in Bulgaria, reporting a particular scenario in which all the dogs involved showed severe clinical signs (diarrhea, vomiting, lethargy, etc.) [33]. Also, poultry is known to be carrier of many pathogens such as *Escherichia coli*, *Campylobacter* spp. and *Salmonella* spp., so there should be support and scientific divulgation to acknowledge backyard farmer on effective veterinary care and disease prevention strategies [30]. Published data further support the zoonotic potential of *Salmonella enterica* subsp. *enterica* serovar Thompson. A 2024 study from Beijing [34] found *S*. Thompson to be the dominant serotype in domestic turtles, with evidence of direct transmission to children. The 9.6% of 480 turtles tested were positive, and over 80% of isolates were multidrug-resistant. Genomic comparison revealed high similarity (<20 SNPs) between turtle and human isolates, reinforcing concerns over pet-to-human transmission [34]. Overall, the available evidence clearly supports the role of companion animals as reservoirs and potential sources of *Salmonella* infection, highlighting their plausible involvement in zoonotic transmission within domestic environments. Our findings are consistent with these data, demonstrating that companion animals (including birds, dogs and cats) can contribute to the persistence and spread of infections within domestic settings. The risk of contracting diseases from pet ownership can be reduced with appropriate preventive measures, but it remains not null, especially in front of the manifestation of specific behaviors (kissing, sleeping in the same bed, sharing food or kitchen utensils) or towards high-risk group members such as children or immunosuppressed patients [6,35]. In the described case, the family’s decision to confine the birds to the aviary and cease consumption of their eggs post-outbreak aligns with recommended preventive strategies. However, the persistent positivity to *Salmonella* in the infant despite antibiotic treatment raises concerns about the effectiveness of current management approaches and the risk of prolonged environmental contamination.

From the perspective of antimicrobial resistance, recent investigations have shown a worrying trend toward multidrug resistance (MDR) in *S*. Thompson strains, with resistant patterns to commonly used antimicrobial classes such as aminopenicillins, tetracyclines, aminoglycosides (streptomycin), and sulfonamides [34,36]. A genomic study conducted in China traced the evolution of co-resistant strains from 1997 to 2020, confirming the presence of stably widespread and genetically conserved clones, even among children and pets [36]. As for the results of in-depth genomic characterization performed on our four selected isolates, MLST analysis revealed that all of them belonged to ST26, which is considered a predominant ST of *S.* Thompson in China [34,36,37,38]. Moreover, from a Bayesian phylogenetic analysis conducted on 136 ST26 *S.* Thompson genomes from different sources and countries, two main separate Chinese clades with one clade including most isolates harboring resistance plasmids and the other one including almost all pan-susceptible isolates, were observed [34,37,38]. In China, the emergence of the concomitant resistance phenotype to ciprofloxacin, third-generation cephalosporins and macrolides in *S*. Thompson has been mainly associated with the spread of an IncC conjugative plasmid, carrying *qnrS1*, *qepA4*, *bla*CMY-2, and *mph*(A), mediating resistance to the above-mentioned molecules [36,37,38]. In Italy a recent survey from Campania region identified *S*. Thompson among antimicrobial-resistant strains circulating in humans, albeit at lower levels compared to *S*. Typhimurium and *S*. Enteritidis [39].

Differently, in the present study almost all isolates were fully susceptible and did not present any AMR genetic basis directly leading to resistant phenotypes, nor any known plasmid replicon potentially related to AMR genes transmission. However, all of them harbored little less than 100 virulence determinants of different VF categories, mostly belonging to the effector delivery-type III secretion system T3SS (TTSS SPI-1 and SPI-2 encode), leading to *Salmonella* invasion in intestinal and extra-intestinal host cells [40], but also genes encoding for fimbrial and non-fimbrial adhesins mediating attachment to the Peyer’s patches and intestinal colonization [41,42]. Most of these genes (located in the same contigs of SPI sequences) have been previously associated with specific SPIs, mediating diverse host–pathogen interactions [43], e.g., the three putative intestinal colonization factors *sinH*, *ratB* and *shdA*, located in the same 25 kb SPI CS54 [44] or the extracellular matrix adhesin encoding the *misL* gene, located within SPI-3 [42] both involved in intestinal colonization and persistence. The presence of such major virulence determinants along with certain SPIs, is consistent with the SPIs and most of the virulence genes previously detected in *S.* Thompson ST26 [34,37,38]. Also in this case, their presence may have favored the intestinal persistence of *S*. Thompson in animals and child.

*Salmonella enterica* serovar Thompson is currently not classified as a high-priority serotype within the European Union, mainly due to its relatively low incidence. According to the most recent European Union One Health Zoonoses Report, in 2023 only 262 confirmed human cases of *S*. Thompson were reported across 13 EU Member States, accounting for just 0.57% of all salmonellosis cases. The only reported outbreak was in the Czech Republic, involving 155 cases, while in Italy only 17 cases occurred [2]. A noteworthy outbreak of *Salmonella* Thompson in Italy happened in 2004, involving a total of 77 children attending nurseries, kindergartens, and primary schools on an island of central Italy [45]. Nevertheless, the incidence of this serotype in human cases at the national level in Italy is quite rare [2,46].

Despite this low prevalence in humans, data from animal surveillance highlights a different pattern. In 2023, in the European Union, *S*. Thompson was the fourth most reported serovar in broilers (5.4%) and was also detected in laying hens (1.4%), indicating its ongoing circulation in the food production chain [2]. These findings suggest that, although rare in clinical cases, *S*. Thompson maintains a presence in animal reservoirs and could represent a latent risk for public health. In the Italian context, *S*. Thompson has been sporadically isolated from wild boars intended for human consumption in the Latium region [47], suggesting possible zoonotic links between wildlife and humans. This association is further supported by another study conducted in northern Italy, which evaluated microbiological contamination in wild boars’ carcasses and organs, finding *S*. Thompson in the cecal content as well as in the lymph nodes, even if sporadically (7% and 3.5% of analyzed organs, respectively) [48]. Leati et al. [5] investigated the epidemiological situation of *Salmonella* in Italy along the poultry production chain within the period 2016–2018, by analyzing data on flock prevalences and serovar distribution collected through the National Control Programmes (NCPs), and data on human isolates collected by the Enteric Pathogen Network (ENTER-NET) surveillance system. Specifically, regarding *S*. Thompson, the authors reported that in breeding hens (*Gallus gallus*), this serovar was detected in 6.1% of cases in 2017 and in 5.5% in 2018, while it was not detected in 2016. In laying hens, the presence of *S*. Thompson accounted for 3.6% in 2016, 3.2% in 2017, and 1.6% in 2018, showing a progressive decrease over time. Finally, in broilers this serovar was the second most frequently detected, with prevalence rates of 15.3% in 2016, 18.7% in 2017, and 8.8% in 2018. Further evidence from southern Italy (Apulia and Basilicata) showed that among 384 chicken food samples collected between 2021 and 2023, *S*. Thompson was detected in only one sample (0.78%), confirming its low, yet persistent, environmental presence [49].

*S*. Thompson has also been implicated in foodborne outbreaks beyond Europe. In South Korea (2018), a large outbreak in ten schools was traced to egg whites used in chocolate cake cream [50]. Similarly, in the United States (2021), 115 cases across 15 states were linked to contaminated seafood processed in a facility with poor sanitation [51]. *S*. Thompson is frequently found in raw eggs, raw chicken, raw red meats, drinking water, dairy products, as well as on eggshell and chicken skin [52]. There is also evidence of the presence of this bacterium in leafy green vegetables [3]. An example can be traced back to the 2004 foodborne outbreak in Norway, where over 100 people were infected after consuming arugula salad imported from Italy. Subsequent detections of *S*. Thompson in arugula by other European countries suggested contamination through non-potable irrigation water [3], highlighting the cross-border implications of foodborne transmission.

In the context of the case examined in the present study, no microbiological analysis was carried out on the food fed to the animals, but according to the parents of the child involved, no raw meat was fed. Although cross-contamination in kitchen during meat manipulation is well recognized as a transmission route for pathogenic bacteria [53], in this case raw meat was not considered to be a more likely source of infection because the lack of direct link to raw meat consumption and animals. The birds, in addition to commercial feed, were also fed with leftover raw vegetables consumed by the family: these foods may also be a potential vehicle for *Salmonella* contamination, for example, in the case of washing with infected water. In this regard, infection through contaminated water is considered unlikely, as no other cases of *S*. Thompson infection have been reported in the area adjacent to the home, suggesting the absence of a widespread environmental outbreak.

In this case study, several potential transmission routes for *S*. Thompson within the household can be hypothesized. One of the main hypotheses is that the infection originated in the birds which, while remaining asymptomatic, may have acted as a reservoir of the pathogen, subsequently transmitting it to the dog and the infant. Transmission could have occurred through direct routes (for example, the dog coming into contact with contaminated fecal material in the garden) or indirect ones, such as cross-contamination: the parents may have handled externally contaminated eggs (on the shell surface) and, despite handwashing, inadvertently introduced the pathogen into the home environment or onto objects used for infant care. Another plausible route involves environmental contamination brought into the home by the dog or the cat, which had free access indoors. However, the initial source of infection in birds remains unclear, as direct contact with wildlife (including large avifauna) is considered unlikely in the context described. Hilbert et al. [54] discussed the transmission of *Salmonella* serovars between rodents and birds, and it is considered plausible, but the issue of the directionality of the transmission remains controversial. In our case, the possibility of rodent-to-bird transmission cannot be excluded, as during the interview with the veterinarians and the parents of the child it was confirmed that rodent burrows were found in the house’s courtyard. However, it is difficult to corroborate this route of transmission due to the absence of direct evidence of the contact between the family’s birds and the rodents and the rapid implementation of rodent control measures. An alternative hypothesis is that the dog was the primary carrier, possibly becoming infected during outdoor activities or walks, and subsequently spreading the pathogen asymptomatically to other animals and the infant. The inclusion of the cat in the cluster remains uncertain, as the only sample collected was taken after the administration of prophylactic antibiotic therapy.

## 5. Conclusions

The increasing prevalence of companion animals in domestic settings underscores the relevance of case studies such as the present one, particularly in the context of public and veterinary health.

*Salmonella enterica* serovar Thompson is a relatively uncommon serotype, and for this reason it often receives limited attention from the surveillance systems.

This study reports a household case of *S*. Thompson infection involving a family and their domestic animals, including ornamental birds, a dog and a cat. The findings suggest that the animals played an active role in the outset and persistence of the infection, highlighting the complexity of potential zoonotic transmission routes in rural household settings.

The strains that were isolated from both the animals and the child were genetically identical, strongly suggesting a common origin of infection. This finding supports the hypothesis that the animals involved (particularly the birds) acted as a reservoir, contributing to recurrent reinfections in the dog and to the persistence of the infection in the child, likely favored by the presence of genetic determinants involved in intestinal colonization and persistence. It is plausible that the pathogen circulated among the birds, the dog, and the infant through direct contact (e.g., exposure to contaminated feces) or indirect pathways (environmental or foodborne contamination). While the primary source of infection remains uncertain, the absence of clinical signs in animals and the symptoms in the infant emphasize the critical role of the domestic environment in the transmission of the pathogen and the importance of preventive measures.

Despite the concerning trend of multidrug resistance reported in *S.* Thompson strains, the isolates analyzed in this study were fully susceptible to the main antibiotic classes tested, and whole-genome sequencing revealed the absence of resistance genes or plasmid replicons potentially associated with AMR gene transmission.

Overall, this case contributes to expand the current knowledge on *S.* Thompson zoonotic transmission dynamics in Italy, especially within domestic settings, also providing expanded data on genomics and antimicrobial susceptibility, and highlights the importance of adopting an integrated approach to health that considers both human and animal health, in line with the One Health framework.

## Figures and Tables

**Table 1:** **Overview of sampling rounds, microbiological testing results and NCBI accession numbers of sequenced samples.** All samples are fecal materials collected from the child and the family’s animals. For each sampling date, the table lists each collected sample along with the species of origin. It is specified whether the fecal sample is a single individual sample or a mixture of faeces in the “Sample type” column. “Pool n.1” and “Pool n.2” means that birds feces were collected as a mixture, from the ground of the aviary, and two distinct pools of feces were sampled on that sampling date. Each sample corresponds to one sampling unit. For each sample, its name (indicated in the “Sample ID” column) and the results of microbiological testing for *Salmonella* spp. (shown in the “Outcome” column) are reported. The outcomes are indicated as follows: (−) negative, (+) positive. The corresponding *Salmonella* serovar is also reported, where applicable (n/a = not applicable). * The avian species represented in the fecal pools included three peacocks (*Pavo cristatus*), four hens (*Gallus gallus*), two guinea fowls (*Numida meleagris*), and one pheasant (*Phasianus colchicus*). § Samples selected for Whole-Genome Sequencing (WGS) and related sequence analysis; their NCBI Accession Number (NCBI Ac. No.) is provided. ^ On 23 April 2025, only one pool of birds’ feces were collected.

Sampling Date	Matrix	Species	Sample Type	Sampling Unit	Sample ID	Outcome	Serovar	NCBI Ac. No.
**7 October 2024**	Feces	Human	Individual	1	SALM_UM_071024 §	+	*S*. Thompson 6,7: k:1,5 O:7 (C1)	SAMN50810632
	Feces	Dog	Individual	1	SALM_VET/1_071024 §	+	*S*. Thompson 6,7: k:1,5 O:7 (C1)	SAMN50810633
	Feces	Birds *	Pool n. 1	1	SALM_VET/2_071024 §	+	*S*. Thompson 6,7: k:1,5 O:7 (C1)	SAMN50810634
	Feces	Birds *	Pool n. 2	1	SALM_VET/3_071024 §	+	*S*. Thompson 6,7: k:1,5 O:7 (C1)	SAMN50810635
**5 November 2024**	Feces	Dog	Individual	1	SALM_VET/1_051124	−	n/a	n/a
	Feces	Birds *	Pool n. 1	1	SALM_VET/2_051124	−	n/a	n/a
	Feces	Birds *	Pool n. 2	1	SALM_VET/3_051124	+	*S*. Thompson 6,7: k:1,5 O:7 (C1)	n/a
**13 December 2024**	Feces	Human	Individual	1	SALM_UM_131224	+	*S*. Thompson 6,7: k:1,5 O:7 (C1)	n/a
**17 December 2024**	Feces	Dog	Individual	1	SALM_VET/1_171224	+	*S*. Thompson 6,7: k:1,5 O:7 (C1)	n/a
	Feces	Birds *	Pool n. 1	1	SALM_VET/2_171224	-	n/a	n/a
	Feces	Birds *	Pool n. 2	1	SALM_VET/3_171224	+	*S*. Thompson 6,7: k:1,5 O:7 (C1)	n/a
**18 January 2025**	Feces	Human	Individual	1	SALM_UM_180125	+	*S*. Thompson 6,7: k:1,5 O:7 (C1)	n/a
**1 March 2025**	Feces	Human	Individual	1	SALM_UM_010325	+	*S*. Thompson 6,7: k:1,5 O:7 (C1)	n/a
**23 April 2025**	Feces	Dog	Individual	1	SALM_VET/1_230425	−	n/a	n/a
	Feces	Birds *	Pool ^	1	SALM_VET/2_230425	−	n/a	n/a
	Feces	Cat	Individual	1	SALM_VET/3_230425	−	n/a	n/a
**14 June 2025**	Feces	Human	Individual	1	SALM_UM_140625	+	*S*. Thompson 6,7: k:1,5 O:7 (C1)	n/a

**Table 2:** ***Salmonella* pathogenicity islands (SPIs) and virulence genes identified in the four *Salmonella* isolates that were in the same contigs**. The outcomes are indicated as follows: (−) negative, (+) positive. * Isolate ID SALM_VET/1_071024 only harboured SPI-2 and the “Not_named” SPI in contig 1. § Only isolate ID SALM_UM_071024 harboured this gene. ^ Genes not harboured by isolate ID SALM_VET/1_071024.

SPIs	Isolate ID SALM_ UM_071024	Isolate ID SALM_ VET/1_071024	Isolate ID SALM_ VET/2_071024	Isolate ID SALM_ VET/3_071024	Virulence Genes	Contig
SPI-2, SPI-5, SP-14, Not_named (Acc.numb. JQ071613)	+	+ *	+	+	*sopA sopE2 steC sseJ steB sifB steA ssaU ssaT ssaS ssaR ssaQ ssaP ssaO ssaN ssaV ssaM ssaL ssaK ssaJ ssaI ssaH ssaG sseG sseF sscB sseE sseD sseC sscA sseB sseA ssaE ssaD ssaC spiC*/*ssaB sifA sopB*/*sigD* ^ *pipB* ^ *sopD2* ^ *slrP* ^, *sspH2* §, *csgC csgA csgB csgD csgE csgF csgG*	1
SP-1, C63PI (SPI-1), SP-13	+	+	+	+	*pipB2*, *avrA orgC orgB orgA prgK prgJ prgI prgH sptP sicP sipA*/*sspA sipD sipC*/*sspC sipB*/*sspB sicA spaS spaR spaQ spaP spaO invJ invI invC invB invA invE invG invF invH sopD*, *mig-14*	2
SP-3	+	+	+	+	*lpfE*, *lpfD*, *lpfC*, *lpfB*, *lpfA*, *misL*, *mgtB*, *mgtC*	4
CS54 (SPI-24)	+	+	+	+	*sseL*, *shdA*, *sinH*, *ratB*, *shdA*	5
SPI-5, SPI-14	−	+	−	−	*slrP sopD2 pipB sopB*/*sigD*	6

## Data Availability

Raw reads can be found in the Sequence Read Archive (SRA) at the GenBank database (NCBI) under the BioProject PRJNA1311533, BioSamples from SAMN50810632 to SAMN50810635.

## Supplementary Materials

The following supporting information can be downloaded at: https://www.mdpi.com/article/10.3390/pathogens14121285/s1, Figure S1: Frequency of major virulence categories and genes in the four *Salmonella* Thompson isolates. Table S1: Antibiotic susceptibility test results and antibiotic resistance genotypes.

## Abbreviations

The following abbreviations are used in this manuscript:

WGS	Whole-Genome Sequencing
NCBI	National Center for Biotechnology Information
IZSLT	Istituto Zooprofilattico Sperimentale di Lazio e Toscana
UOT	Territorial Operative Unit
BPW	Buffered Peptone Water
MSRV	Semi-solid Rappaport-Vassiliadis Agar
XLD	Xylose-Lysine-Desoxycholate Agar
CRRLm	Regional Reference Center for Listeria monocytogenes
CREP	Regional Reference Center
AST	Antimicrobial Susceptibility Testing
NRL-AR	National Reference Laboratory for Antimicrobial Resistance
MIC	Minimum Inhibitory Concentration
SRA	Sequence Read Archive
cgMLST	Core genome Multilocus Sequence Typing
MLST	Multilocus Sequence Typing
SPIs	*Salmonella* Pathogenicity Islands
CGE	Center for Genomic Epidemiology
VF	Virulence factors
AMR	Antimicrobial resistance
ST	Sequence Type
CC	Clonal complex
cgST	Core genome sequence type
MST	Minimum spanning tree
NCPs	National Control Programmes
ENTER-NET	Enteric Pathogen Network
MDR	Multidrug resistance
